# The role of hydrogels in the management of brain tumours: a narrative review

**DOI:** 10.1097/MS9.0000000000001809

**Published:** 2024-02-08

**Authors:** Zahra Anas, Syeda Fatima Saba Hasan, Muhammad Abdul Moiz, Muhammad Abdul Wasay Zuberi, Hussain Haider Shah, Aima Ejaz, Tirth Dave, Muhammad Hasnain Panjwani, Sameer Abdul Rauf, Muhammad Sheheryar Hussain, Radeyah Waseem

**Affiliations:** a Dow University of Health Sciences; b Liaquat National Medical College, Karachi, Pakistan; c Bukovinian State Medical University, Chernivtsi, Ukraine

**Keywords:** Hydrogel, MRI, MRI-monitored therapeutic hydrogel systems

## Abstract

Conventional therapeutic techniques for brain tumours have limitations and side effects, necessitating the need for alternative treatment options. MRI-monitored therapeutic hydrogel systems show potential as a non-surgical approach for brain tumour treatment. Hydrogels have unique physical and chemical properties that make them promising for brain tumour treatment, including the ability to encapsulate therapeutic agents, provide sustained and controlled drug release, and overcome the blood-brain barrier for better penetration. By combining hydrogel systems with MRI techniques, it is possible to develop therapeutic approaches that provide real-time monitoring and controlled release of therapeutic agents. Surgical resection remains important, but there is a growing need for alternative approaches that can complement or replace traditional methods. The objective of this comprehensive narrative review is to evaluate the potential of MRI-monitored therapeutic hydrogel systems in non-surgical brain tumour treatment

## Introduction

Highlights

- Conventional therapeutic techniques for brain tumours, such as surgical resection, chemotherapy, and radiation therapy, have limitations and side effects, necessitating the need for alternative treatment options.
- MRI-monitored therapeutic hydrogel systems show potential as a non-surgical approach for brain tumour treatment.
- By combining hydrogel systems with MRI techniques, it is possible to develop therapeutic approaches that provide real-time monitoring and controlled release of therapeutic agents.
- Hydrogels have unique physical and chemical properties that make them promising for brain tumour treatment, including the ability to encapsulate therapeutic agents, provide sustained and controlled drug release, and overcome the blood-brain barrier for better penetration.
- The integration of MRI techniques with hydrogel systems enables real-time monitoring of tumour progression and treatment response, providing a noninvasive assessment of treatment outcomes.

Hydrogel systems have emerged as promising candidates for the treatment of brain tumours. These three-dimensional networks of hydrophilic polymers possess unique physical and chemical properties especially cross-linking that make them suitable for drug delivery, tissue engineering, and regenerative medicine applications^1^. Hydrogels can be engineered to encapsulate therapeutic agents and provide sustained and controlled release of drugs directly at the tumour site. Additionally, their biocompatibility and tunable properties allow for customization to meet specific requirements in brain tumour treatment. In addition to these aspects Hydrogel was found to be more efficient way than the traditional chemotherapy^2^.

The objective of this comprehensive narrative review is to evaluate the potential of MRI-monitored therapeutic hydrogel systems in non-surgical brain tumour treatment. MRI offers high-resolution anatomical and functional imaging capabilities, allowing for precise monitoring of tumour progression and treatment response. By combining hydrogel systems with MRI techniques, it becomes possible to develop therapeutic approaches that provide real-time monitoring and controlled release of therapeutic agents, while also enabling noninvasive assessment of treatment outcomes.

With the advent of a therapeutic drug delivery system for brain tumours, hydrogels have emerged as a novel therapeutic regime to achieve the optimum delivery of drugs. Hydrogels are three-dimensional polymers composed of hydrophilic polymer chains cross-linking with one another in such a way that it resembles a biological structure in an aqueous solution. These compounds can absorb water but remain insoluble in aqueous or any other solution after forming covalent bonds between the polymeric chains^3,4^. Hydrogels depend on various properties such as biodegradability and biocompatibility for the optimum and safe drug delivery system as they remain exposed to biological cells and tissue for an extended period^3,5^.

Hydrogel formulations are classified based on source derivatives (natural, synthetic, and semi-synthetic), polymeric structures, durability, chemical properties, and network electric charge^5^. For therapeutic and drug delivery purposes, biopolymers can be natural or synthetic based on the derivative material^6^. Natural polymers have better biodegradability and are compatible with body tissues but have poor mechanical properties. Depending upon formulations of hydrogel/biopolymer, natural polymers are of two types protein-based; collagen, gelatin, polypeptide, DNA and polysaccharide-based; hyaluronic acid (HA), chitosan, fibrin, alginate while, synthetic formulations contain polyethylene glycol (PEG), poly vinyl alcohol (PVC), and polycaprolactone (PCL). These synthetic biopolymers have better mechanical properties but poor compatibility with host tissue compared to natural polymers^6,7^.

Hydrogels have mechanical, swelling, and biomedical properties important for tissue engineering and drug delivery systems (DDS)^7,8^. As it is a three-dimensional cross-linking polymer that absorbs and retains water, this ability of hydrogel explains its swelling property. The swelling property is responsible for altering drug release and control. It depends upon external factors such as pH, temperature, and ion concentration. The mechanical properties of hydrogel are important for biomedical applications. These properties such as viscoelasticity, tensile strength, and stiffness determine the polymer’s architecture and release of therapeutic agent for an extended period. The biological properties comprise biodegradability, biocompatibility, non-toxicity, and low immunogenicity^8,9^.

The infiltrative nature of brain tumours such as glioblastoma proves difficult in its resection and treatment with different chemotherapeutic agents^3^. To treat brain tumours, the therapeutic agent must cross the blood-brain barrier. Hence to reduce poor drug penetration, hydrogel systems effectively transfer the chemotherapeutic agents to the site of neoplastic cells by their injectability, structure, and ability to encapsulate the therapeutic agent with reduced toxicity due to swelling and degradable properties. The use of nanoparticles in hydrogels for optimum drug delivery arises due to the incompatibility of the drug with hydrogel systems. The hydrophilic nature of hydrogel and the hydrophobic property of drugs create barriers to drug delivery. Nanogel overcomes this by encapsulating the drug and is compatible with hydrogel. This combination proves significant as it helps in controlling drug release. Nanogels provide the following advantages: site-specific drug therapy, passively-controlled drug release, stimuli-responsive drug therapy, and detoxification. Nanogels can also decrease the volume of tumour cells migration outside the cortex into the hydrogel system loaded with chemotherapeutic drugs^3,10,11^. In addition, hydrogel systems can detect tumour sites when filled with radioisotopes and provide effective radiotherapy^10,11^.

**Figure 1:**
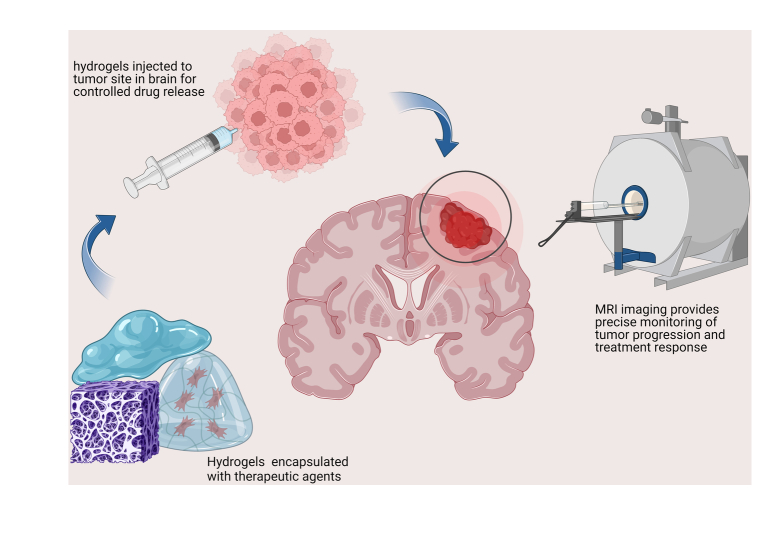
The process of hydrogel application, loaded with therapeutic drug, in treating brain tumours.

Brain tumours are routinely treated through surgical resection, chemotherapy, and radiotherapy. Bastiancich *et al*.^12^ developed lipid-based nano capsules in a hydrogel loaded with drugs that shrink tumour cells and are more effective than the drug itself. Another hydrogel system with cancer cell sticky (CSH) hydrogel helps decrease invasiveness by trapping such cells^13^. Other hydrogel systems for brain tumours with different combinations are polymeric micelles, magnetic nanoparticles, alginate nanogels loaded with gold particles, and microspheres^3,13^. Figure 1 briefly explains the process of hydrogel application, loaded with therapeutic drug, in treating brain tumour.

## Role of hydrogels in treating different types of brain tumours

Glioblastoma multiforme (GBM) is a grade IV Astrocytoma of glial cell origin. It is highly aggressive and has a poor prognosis^14^. Despite the traditional therapies, there are increased chances of relapse, adverse systemic events of chemotherapy, and often chemotherapeutic agents have less effect due to poor penetration and efficacy. Different methods for efficacious drug delivery, such as postoperative biodegradable Gliadel wafers usage in the resected cavity for better drug delivery, have not yielded better results. Hence, a novel therapy for optimum drug administration in the form of hydrogels is introduced^14,15^

Bouche and colleagues proposed the utilization of selenium-based hydrogel laden with Quinostat, a highly effective anti-(GBM) drug, and radioactive material. The study observed radiation-based drug release and radiotherapy. The in-vivo hydrogel infusion in mice showed a reduced tumour relapse rate and decreased progression without any reported systemic adverse events. In addition, the hydrogel consisted of radio-opaque gold nanoparticles to observe the long-term drug release and hydrogel degradation^15^. Another similar study by Zhao and colleagues suggested a hypothesis of using photopolymerizable hydrogel infused with paclitaxel anti-(GBM) drug with nanoparticles injected in postoperative mice resection cavity. It demonstrated a 50% increase in long-term survival compared to the control group mice. The only challenge faced was microglial inflammation due to the polymer used, but it was also beneficial in removing the tumour debris^16^. Sayiner *et al*.^17^ designed a thermos-reversible hydrogel that consisted of temozolomide encapsulated in nanoparticles with PGLA hydrogel. They observed a successful controlled and sustained drug release for 60 days. Hydrogels also play a role in increasing efficacy and decreasing the resistance against chemotherapeutic drug temozolomide^18,19^. Wang *et al*.^19^ utilized a liposome-based nanogel that encapsulated temozolomide and erastin that causes iron-based apoptosis of temozolomide-resistant tumour cells. The role of hydrogels in glioblastoma ranges from decreasing the relapse rate, imaging the tumour cells, drug control, drug release, and treatment of drug-resistant tumours. Although these hydrogel formulations help hydrophobic drug solubilization for better drug delivery and prevent burst release of encapsulated drugs, they also can be used in the immunotherapy treatment of cancer. In immunotherapy, thermo-sensitive hydrogels help in better T-cell penetration inside the tumour, creating a cytotoxic effect toward tumour cells^20^.

Astrocytoma is a primary glial cell tumour that originates from astrocytes and has high-grade and low-grade forms. Astrocytoma’s mainstay of treatment is surgical resection and adjuvant chemotherapy with postoperative radiotherapy^21^. High-grade IV astrocytoma is known as Glioblastoma multiforme. Hence most hydrogel applications are similar to the ones used in treating Gliomas. Hydrogels can play a role in tissue engineering and cell culture, especially for tumour cells to understand their behaviour (migration, cellular differentiation, angiogenesis), and different biomarkers can be assessed to aid in treatment^22^. Jogalekar and colleagues assessed the cell morphology of two malignant Astrocytoma cell lines in monolayer cell culture and a hydrogel scaffold. The hydrogel scaffold provided a better tissue microenvironment than its counterpart^21^. Another study by Jogalekar *et al*.^23^ extracted total RNA from hydrogel-based cell culture to assess the genes involved in the pathogenesis of astrocytoma and note their upregulation; this proved to be important in the future of glioma therapy. Despite all the breakthroughs in hydrogel use for the treatment of GBM, the clinical translation of such biotechnology has been poor as there are no clinical trials conducted. The in-vivo and in-vitro studies on mice and tissue-engineered tumour cells show a great success, but the safety of using hydrogel formulation directly on the human brain should be evaluated^20^.

Apart from the vast applications of hydrogels in glial cell tumours, they can be applied to other areas of Central nervous system tumours, such as meningiomas. These tumours arise from meningothelial cells of the brain and spinal cord. They are low grade and high grade according to WHO classification. They are treated by surgical resection with coagulation of dural attachment, depending on the tumour grading^24^. High-grade meningiomas can cause vessel injury during resection surgery because they encase the arteries and veins^25^. Yang and colleagues proposed that thermo-sensitive polyester hydrogel polymers can be utilized in the embolization of arteries to improve surgery outcomes of different tumours such as meningioma, uterine fibroids, and hemangioma. This biodegradable polymer could achieve short-term embolization of the pharyngeal artery in a swine. Nevertheless, the thermogel demonstrated weak strength, and the artery was recanalized after 1 h; hence this proved challenging in treatment and require further research^26^.

Ependymoma is a neuroepithelial tumour of the central nervous system derived from ependymal cells of the ventricles and spinal canal. This most common childhood central nervous system tumour can be supratentorial, infratentorial, and spinal in nature, depending on the site of origin. The mainstay treatment is gross surgical resection of the mass due to clear demarcation of the tumour mass with postoperative radiotherapy. Chemotherapy is avoided in children due to high cytotoxicity^27^. Hence an effective chemotherapeutic drug delivery system to treat such tumours after resection to prevent systemic cytotoxicity is required. The clinical trials for supratentorial ependymoma treatment are ongoing, where in-vitro and in-vivo hydrogel-based drug delivery in mice is being assessed^28^. Hydrogels are also used as tissue scaffolds for tissue microenvironment assessment and different targeted therapies for ependymal tumours and medulloblastoma in children^29^. This scaffold is used for many aspects, such as to assess cell adhesions and various proteins to understand the metastatic nature of such tumours. Such tissue engineering is necessary because many therapies are successful in in-vitro models but fail in in-vivo animal models. Therefore appropriate three-dimensional hydrogel polymers that mimic the tumour cell environment can help in pre-clinical testing and the response of anti-cancer drugs^30^.

Other discrete tumours, such as schwannoma and pituitary adenoma, have limited hydrogel use due to a lack of research conducted. However, hydrogels have been used as durant seals to prevent Cerebrospinal fluid leakage in durotomy when removing spinal schwannoma^31,32^. In trans-sphenoidal surgery for pituitary adenoma resection, Polyethylene Glycol (PEG) is used as a dural sealant to avoid defects and cerebrospinal fluid leakage. Clinton and colleagues conducted a single-centred retrospective study comparing PEG hydrogel, collagen matrix, fat grafts, and lumber drain placement use in preventing postoperative cerebrospinal fluid (CSF) rhinorrhea. This study observed that a combination of PEG hydrogel and collagen matrix was effective in controlling CSF leakage and more cost-effective with low morbidity compared to fat grafts and lumbar drain placement^33^. A randomized controlled trial in a multi-centred study showed pituitary adenoma treatment with subcutaneous octreotide hydrogel implant in patients with acromegaly. It proved more efficient than the frequent depot intramuscular and subcutaneous injections which are uncomfortable. This prolonged treatment interval provided a slow controlled drug release for 6 months and improved compliance^34^. Although the role of hydrogels in therapy, diagnosis, and tissue engineering for brain tumours is promising, further research and trials are required to understand and implement such hydrogel applications (Table 1).

**Table 1:** Treatment and hydrogel application for brain tumours

Tumour type	Treatment and hydrogel applications	References
Glioblastoma multiforme	Selenium-based hydrogel with quinosta Photopolymerizable hydrogel with paclitaxel Thermoreversible hydrogel with temozolomide Liposome-based nanogel with temozolomide and erastin for temozolomide-resistant tumours Immunotherapy with thermo-sensitive hydrogels	^14–17,19,20^
Astrocytoma	Similar applications to GBM treatment Hydrogel scaffold for cell culture and tissue microenvironment assessment Gene expression analysis for pathogenesis of astrocytoma	^21–23^
Meningioma	Use of thermo-sensitive polyester hydrogel polymers in arterial embolization	^26^
Ependymoma	Ongoing clinical trials for hydrogel-based drug delivery Tissue scaffolds for microenvironment assessment and targeted therapies 3D hydrogel models for pre-clinical testing	^27–30^
Schwannoma	Used in dural sealing and CSF leakage prevention PEG hydrogel and collagen matrix combination effective for CSF leakage control.	^31–33^
Pituitary adenoma	Subcutaneous octreotide hydrogel implant for pituitary adenoma treatment	^34^

3D, three dimensional; CSF, cerebrospinal fluid; GBM, Glioblastoma multiforme; PEG, polyethylene glycol.

## Role of MRI monitoring in therapeutic hydrogel systems

The radiological modality of choice for a successful diagnosis of a brain tumour is Magnetic Resonance Imaging with contrast (gadolinium enhanced)^35^. Since there are no specific pathognomonic features to differentiate between primary and secondary tumours, MRI does not tell the place of origin. However, it is very specific for tumour detection and its precise location^36^. Even though, contrast MRI has a key role in the diagnosis of brain tumour, it only provides an approximate of tumour grade and histology, which are equally important to determine before management is planned. In recent years, advances in types of MRI, like diffusion weighted imaging (DWI) and perfusion weighted imaging (PWI) have made it possible to procure information vis a vis cellular matrix and relative regional cerebral blood volume which makes the diagnosis precise and more accurate^37^.

Hydrogels are hydrophilic polymer networks which are being suggested for a number of biomedical interventions because of their rheological properties. They are drug delivery systems which can be injected into the tissues and allow sustained release of therapeutic agents^38^. For brain tumours, chemotherapy, which is the treatment of choice in most cases is given through hydrogels which provide prolonged exposure to the effective dose after only one intracerebral injection^39^. Certain hydrogels are naturally fluorescent; some are encapsulated with contrast materials and are traceable on MRI scans while others are designed to produce contrast in the body via chemical exchange saturation transfer (CEST) technique^40^. After the hydrogel is administered, MRI can be effectively used to noninvasively monitor the in-vivo release of proteins and check if the therapy is working^39,40^. The advantage of using this system is that hydrogels are carefully controlled, and they offer diagnosis, drug delivery and monitoring of therapeutic response in a single platform.

While some hydrogels are naturally imageable due to their intrinsic fluorescent properties, others require incorporation of contrast agent into the polymer to make them visible on MRI imaging^41^. Contrast materials are therefore either physically or chemically attached to the hydrogel compound. Physically loaded contrasts are retained in the hydrogel via ionic bond, hydrogen bond, coordination bond, or covalent cross links. Hydrogels are incubated with contrast material to allow the formation of these bonds either in a network or a capsule^42^. Chemical insertion of contrast is done via grafting of high energy covalent bond with the hydrogel polymer. Compared with the physical loading, the displacement of contrast from the hydrogel is less likely and so it is more accurate in quantifying the remaining hydrogel^41,43^.

## Safety and biocompatibility of hydrogels

Hydrogel-based tools are now being frequently used in biomedical applications such as in delivery of drugs, to initiate treatment of disease in any organ of the body like that brain, kidney, intestines, eye etc^20^. To analyze whether the use of these polymers in an already ill patients is safe, the biocompatibility of hydrogels after they are administered is important to determine. A study examined the in-vivo biological effect of a hydrogel post-brain injection in mice and reported no damage to the surrounding brain parenchyma^44^. Research on the outcome of using hydrogel to deliver chemotherapy in patients with GBM concluded that hydrogels demonstrate in-vivo biocompatibility and there were no significant side effects of this method of drug delivery^45^. Hydrogels have compressive and mechanical properties which can be utilized in wound dressing if there is satisfactory evidence that they do not trigger an unwanted response in the body. According to a study, hydrogels demonstrated not only lower inflammation but also rapid healing rate for in-vivo wound repair in rats^46^. An investigation conducted on rats using hydrogel implants which have been proposed as a treatment for neurodegenerative disorders or permanent brain injury reported no apparent inflammatory, immunogenic, or tumorigenic side effect as a consequence of implants^47^. These examples show that hydrogels are not only effective and efficient but also biocompatible and safe to use.

Hydrogels are administered via percutaneous injection which are extremely painful. There is also a risk of injection site reaction and entry of bacteria through the puncture which results in infections. To avoid this, the new micro needle transdermal approach is being deployed. Micro needles are minimally invasive, painless, and easy to use. They are smaller (about 1 mm) than traditional injection needles which are at least 4 mm in length and pierce only the dermis without any damage to the nerves and vessels of the dermis^48^. Some hydrogels used in the treatment of tumours lead to a short term yet a severe photo thermal effect. Initially the hydrogels do not spread to the whole tumour but produce local hyperthermia. This high temperature causes inhibition of the rest of the tumour due to heat. Even though, this rise in temperature is therapeutic, but it is unpleasant and so it can be avoided by combining the hydrogel with other treatment modalities like photodynamic therapy, chemotherapy, vascular rupture therapy, or catalytic therapy^49,50^. Owing to their small size, hydrogels have a burst release that is a lot of drugs is released in a very short time which can cause acute local and systemic toxicity. Since an appreciable amount of drug is lost, the therapeutic efficacy declines over days^48,51^.

Generally, hydrogels rarely present with adverse effects but their potential side effects are being explored to optimize their use. A study which focused on investigating the immune compatibility and non-thrombogenicity of gelatin-based hydrogels reported that there was no complement activation or neutrophil reaction as well as no incidence of bleeding, vessel damage or thrombosis in hydrogels which were coated with a suitable substance for example Low-density lipoprotein for cardiac implants. Mild follicular hyperplasia in lymph nodes was observed in the nice receiving non coated hydrogels. This comparison shows that to reduce the risk of immune reaction and lymphadenopathy, hydrogels should be encapsulated with a material suitable for the tissue being treated^52^. Hydrogels are being increasingly employed to treat degenerative disease like Alzheimer’s but on exposure to reactive oxygen species, the properties of hydrogel delivered stem cells are altered which means they lose their regeneration potential and cause treatment failure^53^ One problem with hydrogel is that they can be non-adhesive [alginate, carboxymethyl cellulose] or totally biodegradable. This affects their long-term use as controlled interventional agents because it is important that they stick within the body and are not broken down by normal physiological processes. This problem is most pronounced when they’re used as a wound dressing. To manage this issue, hydrogels are covered with secondary adhesive substances to ensure they remain where they are needed^54^.

One major concern governing the use of hydrogels is the potential immune response they can trigger within the body which could aggravate the disease condition instead of mitigating it. Not all the time, the immune reaction to a hydrogel is undesirable as in immunotherapy, like in the case of GBM [a lethal malignant brain tumour] where the hydrogel is designed in a way to produce specific antigen receptor macrophages which surround the cavity and kill the tumour cells in order to prevent relapse after resection^55,56^. However, on most instances the immunostimulant property of hydrogels can be a menace leading to treatment failure. To avoid this, hydrogels are combined with different substances which reduce their immunogenicity or are encapsulated with materials that are non-immunogenic to protect them from destruction before they reach the place where the intervention is required. Hydrogels are also coated with anti-inflammatory drugs to reduce inflammation and injury such as to microglial tissues post an intracerebral injection of hydrogel^57^. Hydrogel-based compounds are now incorporated with different kinds of molecular and cellular signals to control the immune response instead of completely curbing it off. This is best shown in wound healing where there is calculated immune simulation according to stage of healing^58^. This gives a prospect to build on in future and avoid unrequited reaction of our body to use of hydrogels in biomedicine.

## Conclusion

There are significant challenges and limitations faced in treatment of brain tumours through conventional therapeutic techniques such as surgical resection, chemotherapy and radiation therapy. This necessitates the need of alternative treatment options for management of brain tumours that have lesser side effects and are lesser invasive.

Unique physical and chemical properties of hydrogels have signified them as a promising treatment option in future for treatment of brain tumours. They can encapsulate different therapeutic agents and provide a much sustained and controlled drug release then administering drugs directly by conventional drug routes. Furthermore they can overcome the blood-brain barrier and provide better penetration.

Integration of MRI enhances the effectiveness of hydrogel systems by providing high-resolution anatomical and functional imaging and to monitor their treatment response.

Safety and biocompatibility of hydrogel systems have been well established through many clinical and pre-clinical tryouts. Although still a need to ascertain their safety needs to be assured to prevent any potential side effects that can result from their use.

In conclusion use of MRI-monitored hydrogel system is promising in future treatment of brain tumours that provides an advanced noninvasive treatment approach which can greatly reduce morbidity and mortality resulting from surgical resection.

## Ethical approval

Not applicable.

## Consent

Not applicable.

## Source of funding

Not applicable.

## Author contribution

S.F.S.H.: conceptualization, drafting the work, final approval, and agreement to be accountable for all aspects of the work. Z.A. and M.A.Z.: drafting the manuscript, final approval, and agreement to be accountable for all aspects of the work. M.A.W.Z.: drafting the manuscript, final approval, and agreement to be accountable for all aspects of the work. H.H.S. and T.D.: drafting the work, final approval, and agreement to be accountable for all aspects of the work. A.E. and T.D.: drafting the manuscript, final approval, and agreement to be accountable for all aspects of the work. M.H.P.: drafting the work, final approval, and agreement to be accountable for all aspects of the work.

## Conflicts of interest disclosure

None declared by the authors.

## Research registration unique identifying number (UIN)

1. Name of the registry: Not Applicable.
2. Unique Identifying number or registration ID: Not Applicable.
3. Hyperlink to your specific registration (must be publicly accessible and will be checked): Not Applicable.

## Guarantor

Muhammad Abdul Wasay Zuberi.

## Data availability statement

It will be available upon reasonable request.

## Provenance and peer review

Not commissioned, externally peer-reviewed.
